# Transvaginally Adjustable Apical Suspension and Compartment-Specific Tensioning in Vaginal Natural-Orifice Transluminal Endoscopic Surgery Sacrocolpopexy: Cadaveric and Live Patient Experience

**DOI:** 10.1089/gyn.2023.0120

**Published:** 2024-04-15

**Authors:** Dani Zoorob, Eric Shuffle, Jay Matkins, Oz Harmanli

**Affiliations:** ^1^ UroGynecology Division, Department of Obstetrics and Gynecology, Louisiana State University Health Sciences Center at Shreveport, Shreveport, Louisiana, USA.; ^2^ Department of Obstetrics and Gynecology, ProMedica Health System, Toledo, Ohio, USA.; ^3^ Department of Obstetrics and Gynecology, Atrium Health, Charlotte, North Carolina, USA.; ^4^ Department of Obstetrics, Gynecology, and Reproductive Sciences, Yale School of Medicine, Yale University, New Haven, Connecticut, USA.

**Keywords:** mesh, natural orifice, prolapse, sacrocolpopexy, tensioning, vaginal, vNOTES

## Abstract

**Objective:**

This article provides a systematic approach to performing a vaginal natural-orifice transluminal endoscopic surgery (vNOTES) sacrocolpopexy (SCP) to create an anatomically aligned vaginal axis, an intraoperatively adjustable apical suspension, and variable compartment tensioning.

**Methods:**

The technique presented for vNOTES SCP focuses on: (1) retroperitoneal tunneling; (2) direct sacrum access below the S-1 level, using uterosacral-ligament guidance; (3) transvaginal tensioning of the mesh to ensure both adequate vaginal length and cuff elevation using the DZOH apical-suspension technique; (4) circumvention of intrapelvic laparoscopic suturing; and (5) near-total peritoneal coverage of the mesh arms.

**Results:**

This detailed description of a successful novel technique to perform vNOTES SCP was based on cadaveric experience as well as in live patients that is reproducible on living patients.

**Conclusions:**

This apical suspension technique for vNOTES SCP may be a viable, reproducible, safe, and efficient transvaginal alternative to the commonly practiced minimally invasive approaches that involve abdominal-port placements. (J GYNECOL SURG 40:116)

## Introduction

Sacrocolpopexy (SCP) is one of the most commonly performed procedures to address apical pelvic-organ prolapse (POP) in women. Conventionally, this was performed through a larger laparotomy incision, offering access, safety, and mesh coverage. With the advent of minimally invasive techniques, wherein laparoscopic ports were placed across the abdomen, this potentially complex procedure became more popular as recovery became swifter, the need for larger incisions was circumvented, and safety was enhanced. However, when considering cost-effectiveness, operative-theater accessibility, and ease-of-recovery from discomfort and rapidity standpoints, completing the SCP entirely vaginally (sacral dissection, sacral-mesh attachment and tensioning, and peritoneal coverage of the mesh) may be a viable alternative that also offers patients the benefits attributed to vaginal and natural-orifice advanced laparoscopic surgeries (per McGee et al., 2006).^1^ These include superior cosmesis, reduced risk of abdominal-hernia formation and morbidity, less postoperative discomfort, and faster recovery.

Many pelvic surgeons already perform various steps of SCPs vaginally, including hysterectomy, dissection of rectovaginal and vesicovaginal planes for mesh attachment, and finally, vaginal attachment of the mesh.^2,3^ Due to innovative port designs currently on the global market, vaginal natural-orifice transluminal endoscopic surgery (vNOTES) has become more feasible and desirable. This has expanded the potential for performing various gynecologic procedures while performing laparoscopy solely through the vagina. This setup helps eliminate the needs for abdominal port entry, robotic availability and assistance, and turnaround time required for setup for various procedures, including SCP.^4^

The benefits of this approach may include circumventing the confines of abdominal adhesions and abdominal-wall mesh, offering an alternative surgical route when abdominal entry is limited and providing new surgical opportunities at institutions without robotics, while also limiting operating-room expenses associated with surgery that may be robotically related or due to having multiple setups. Such considerations have been reported by Liu et al., who confirmed the feasibility of SCP performed through a vaginal port^4^; whereas Chen et al. demonstrated shorter operating times relative to single-incision abdominal access.^5^

Access to the sacrum through the vaginal port has also been described for various techniques, including (1) incising the peritoneum to access the anterior longitudinal ligament (ALL) and (2) tunneling, whether started retroperitoneally within the pelvis or from outside the pelvis (referred to as pure *extraperitoneal pelvic*).^6,7^ All techniques require layered dissection while remaining directly underneath the peritoneal layer to protect the underlying structures and avoid injury. However, the former enables dissection of the peritoneum off the pelvic sidewall to be approximated thereafter as needed.

Care to avoid vital structures is necessary, especially because extensive laparoscopic suturing is required to cover the mesh and sacrum fully. The latter, which involves the tunneling technique, facilitates mesh placement as it is simply pushed through a tunnel, which may reduce the need for additional time for suturing and thus potentially avoid laparoscopic suturing–related injuries. Planning the track of the tunnel before initiating dissection, repeated intraoperative assessment of the locations of the instruments relative to the target, and avoiding deviation help add safety to this technique. Retroperitoneal bleeding needs to be assessed and hemostasis should be ensured before and after mesh layering.

Combination techniques have also been described wherein a tunnel is made and the peritoneum covering the ALL is incised to enable suturing, which may reduce the time needed for this surgical step. However, the risks associated with suturing and time requirements still exist, as the incised area will require approximation to ensure that the mesh is retroperitonealized.^8^ Various ALL exposure permutations and modifications of the mesh attachment have been described.

The current authors' cadaveric experience and review of vNOTES literature suggests the following steps as challenges: (1) mesh attachment to the ALL; (2) ensuring adequate vaginal length, especially when it may not be adjustable once the mesh has been precut or attached even when direct measurement is performed; (3) the suspension may not allow compartment-specific guided tensioning (increased or decreased tensioning or support for 1 compartment versus another); (4) tensioning the sacral end of the mesh may be difficult due to poor access or ability to suture; and (5) coverage of the mesh with peritoneum may be limited or challenging due to difficulty in suturing.^9^

This article methodically describes the key steps to perform a vNOTES SCP successfully, safely, and efficiently, using a technique that produces (1) a customizable vaginal length, (2) adjustable apical tensioning, (3) anterior and posterior compartment-specific suspension variation, and (4) retroperitoneal containment of the mesh, while (5) avoiding intrapelvic laparoscopic suturing. There is also a description of the DZOH apical tensioning technique, which assists with intraoperatively adjustable vaginally guided cuff elevation. The steps described are suggestions and demonstrate how the current authors performed for their live and cadaveric cases.

## Methods

### Institutional review board approval

Institutional review board approval for this work was obtained from ProMedica, Toledo, OH. (IRB #23-015)

### Patients and materials used

Both cadaveric and live patients were used to simulate and reproduce successfully the steps described by 2 seasoned board-certified urogynecologists. Informed consent was obtained from the living patients. A Gelpoint V-Path™ (Applied Medical, CA, USA), a gel cover with port attachments, and an Alexis Retractor™ (Applied Medical), a plastic-sheath double-ring retractor, were used in the completion of the procedures described while permitting the use of standard laparoscopic instruments and establishing a pneumoperitoneum transvaginally.

### Surgical procedure

The first step requires the vaginal hysterectomy to be completed, which may be performed through the vNOTES route with the vNOTES port kept in place upon completion, enabling an easy transition into the SCP component of the surgery. Alternatively, a conventional vaginal hysterectomy may be performed, followed by placement of the vNOTES port. The port's internal ring should ideally be placed distal to the ischial spines and as far away from the sacrum as possible. This will enable easy manipulation of the rectum, if needed, and will reduce the tension on the pelvic portion of the peritoneum. Additionally, the sutures attached to the distal portion of the uterosacral ligament (USL) are maintained throughout the case, given that firm traction applied at 1 point during the case will help identify the track of the USL proximally close to the sacrum. This will help suggest the level at which the dissection of the ALL at the sacrum may be made, enabling an anatomically aligned vaginal axis when the apical mesh is attached. The steps of this procedure can also be performed following successful transperitoneal entry into the vaginal vault post hysterectomy.

**FIG. 1.**
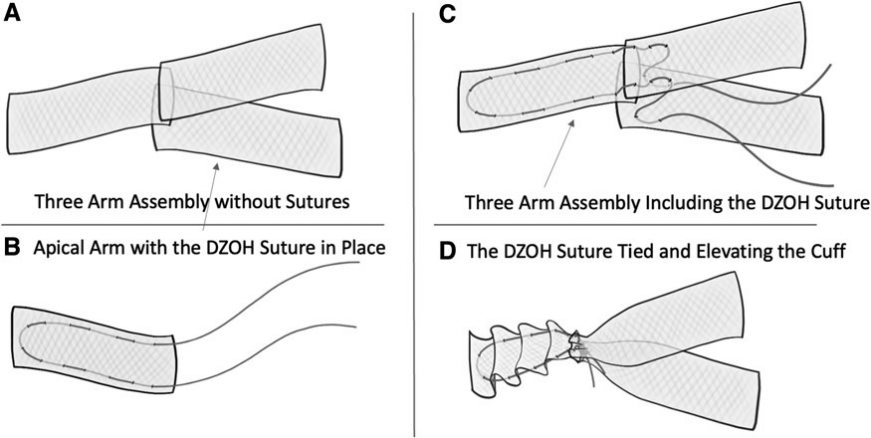
Mesh setup. **(A)** Without the Prolene Suspension Suture.^®^ **(B)** Apical arm with the DZOH suture in place. **(C)** Mesh arm design with DZOH Prolene suture placed on the apical mesh arm and threaded into the anterior and posterior arms. **(D)** DZOH suture tied and elevating the cuff simulating vaginal suspension.

The transvaginal mesh can be prepared using a precut (Y) mesh or a single-layer type-I polypropylene mesh piece (measuring at least 12 x 12 cm). If a single piece is used, the mesh is cut into 3 separate pieces, each measuring ∼4 x 10 cm. To enable adjustment of the apex and adequate tensioning, a 0-Prolene suture (measuring at least 20 cm long) is threaded into the sacral arm in a U shape. The DZOH vNOTES apical tensioning technique requires folding the sacral mesh into an accordionlike structure. Then, a suture is threaded from 1 side of the mesh accordion to the other side; the needle is then spaced one 1 cm away from the exit site and then threaded into the mesh in the opposite direction. This will enable a distinctive U-shape threading where both suture edges will emerge from 1 mesh end, while the angle within the suture is on the opposite side of the mesh. This angle, or the bottom of the U, will be the proximal mesh end that will be attached to the ALL, whereas the distal arm of the mesh with the emerging 2 sutures will be attached to the other 2 mesh arms (each arising from the anterior and posterior compartments). This suture will be used later in the procedure to generate the necessary tension on the mesh while elevating the cuff and connecting all 3 arms together permanently (Fig. 1).

With the patient in a maximum Trendelenburg position and the vNOTES port in place, the bowel is mobilized above the pelvic brim, using graspers. A moist laparotomy towel may be considered for mobilization, if needed. Once the surgical field is clear, the ideal location for tunnel initiation is delineated. This is the middle part of the pelvis, slightly right of midline, at the level of the ischial spine, in an area devoid of vasculature that is medial to the uterosacral ligament and lateral to the rectum. At this level, the right ureter will be lateral to the uterosacral ligament and safe during the dissection.

**FIG. 2.**
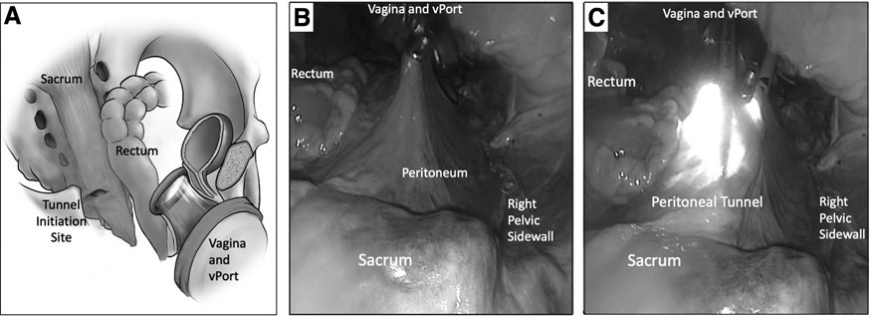
Views of the pelvis with **(A)** tunnel-initiation site, **(B)** instruments in the tunnel dissecting from the direction of the vagina to the sacrum without camera lighting up the tunnel, and **(C)** tunnel with laparoscopic instruments and the camera within. vPort, virtual port.

Accordingly, a 3-cm horizontal incision, started medially and spread laterally to the rectum toward the right side of the pelvic sidewall is made using cold-knife shears. This helps limit uncontrolled extension of the incision; thus, potentially protecting the rectum.^10^ Displacing the rectum to the contralateral side of the pelvis upon initiation of tunneling may protect the rectum further.^11^ The current authors' team prefers to use the “Tent and Tunnel” technique. Tenting the peritoneum upward toward the abdominal wall enables tunneling, improving visualization of the nearby vasculature while enabling visualization of the peritoneal edge and structures within it (Fig. 2). Tunneling can be made using cold scissors and a Maryland grasper and needs to be maintained very close to the peritoneum. The instruments must lead into the tunnel while the camera is advanced behind them, as the surgeon is closely monitoring the dissection being performed.

The direction and angle of the dissection are defined after delineating the potential site of attachment. To identify the most anatomically aligned attachment site (∼ S-1–S-2 level, below the sacral promontory, and away from the common iliac vessels), both USLs are placed under visible tension through strong external traction on sutures attached to the distal ends of the ligaments. The arcs of the ligaments are visualized and their confluence at the sacrum can suggest the level for dissection and mesh attachment. The dissection is guided and angled to the suggested site thereafter.

**FIG. 3.**
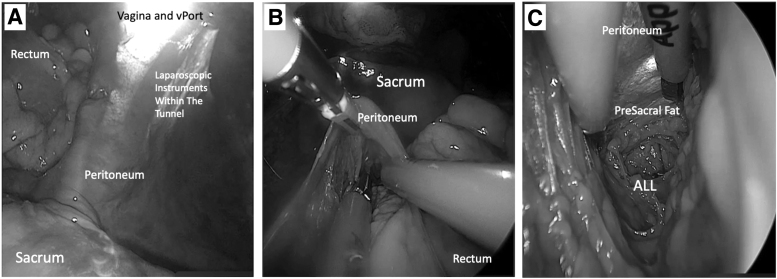
Tunnel view in proximity of the sacrum. **(A)** Cephalad view of the instruments reaching the sacrum. **(B)** Surgeon view of instruments in the tunnel looking toward the sacrum, **(C)** anterior longitudinal ligament (ALL) exposed and seen within the tunnel.

When the sacrum is both identified visually and confirmed tactilely, using a Kittner, a space along the ALL is delineated to be exposed for mesh attachment. The surgeon needs to be aware of the following structures present in the presacral space: common-iliac, middle-sacral, and inferior mesenteric arteries; ureters; superior hypogastric plexus; and hypogastric nerves at all times.^11,12^ However, due to the axis of dissection, this surgical route may enable increased safety by generally being remote from the critical larger vessels (dissection site is below the level of the promontory) and structures investing the sacral region. Using an endoscopic Kittner, tracking along the firm sacral bone in a vertical manner can help mobilize the fat off the ligament while safeguarding the middle-sacral artery (Fig. 3). Note that less fat may be encountered below the level of the promontory.

**FIG. 4.**
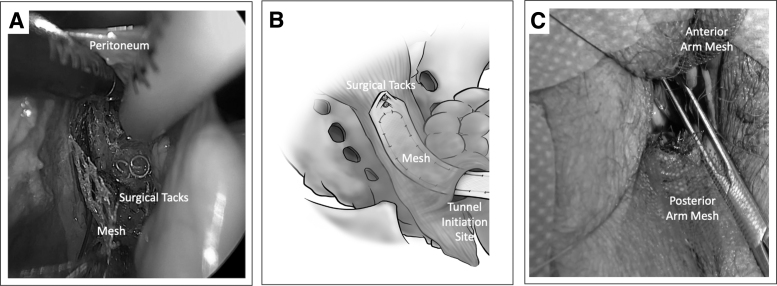
Peritoneal tunnel showing **(A)** tacks on anterior longitudinal ligament affixing the apical mesh in place. **(B)** Sketch depicting the mesh with the DZOH suture laying against the sacrum prior to tensioning. **(C)** Mesh attached to the anterior and posterior vaginal walls.

Thereafter, the mesh with the Prolene^®^ suture attached is introduced transvaginally through the larger trocars on the vNOTES port, with the free suture ends remaining distal and pointing away from the sacrum. The suture ends ideally should remain emerging from the trocar until removal of the vNOTES port. Apical traction on the tip of the mesh can help maintain the suture in place while advancing the mesh, resulting in reduced bunching and resistance. A surgical tacker (such as the Protack,™ Medtronic, Minneapolis, MN, USA), which offers efficient controlled attachment, can be used to connect the mesh to the ALL under direct visualization while avoiding the middle-sacral artery (Fig. 4). Two-to-three tacks may be applied to affix the mesh to the ALL. After ensuring that the mesh and peritoneum are laying as flat as possible, the vNOTES port may be removed. The sutures attached to the USL should be trimmed at this point.

The mesh attachment on the anterior vaginal wall is then initiated by everting the anterior vaginal wall outward with the help of Allis clamps. Copious hydrodissection from the intrapelvic side (not the vaginal-skin side) enables identification of the fibromuscularis layer, thus facilitating the dissection of the bladder tissue. This is carefully released off the anterior vaginal wall starting from the anterior cuff edge for ∼5 cm while avoiding dissection near the trigone. The same technique is performed on the posterior vaginal wall, while preferably avoiding the perineal body. Some surgeons may want to attach the mesh to the perineal body to reduce perineal descent.

Then, the anterior and posterior mesh arm pieces should be attached to their respective vaginal walls with at least 6 interrupted sutures on each side. The choice of sutures used is based on the surgeon's preference. As both mesh pieces are precut long, several cm of mesh will be hanging freely off both anterior and posterior cuff edges. These excess mesh edges must be trimmed, leaving, on average 2 cm, protruding past the vaginal-cuff edge. When offsetting support or tension for a specific compartment, variability in mesh length excess past the vaginal end helps redistribute the tension and the angle of support when all three mesh arms are connected to one another and tensioned.

**FIG. 5.**
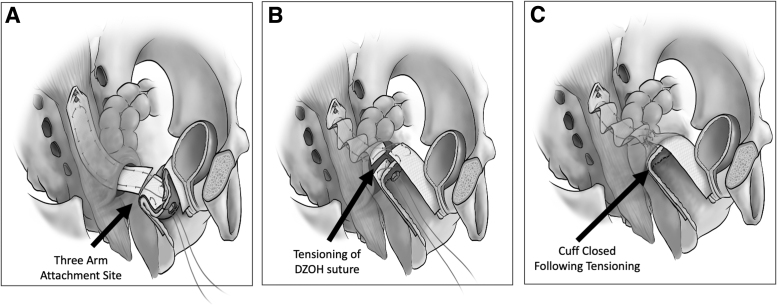
Sketch depicting the progression of the 3 arms when connected. **(A)** Apical arm with the DZOH apical suspension suture prior to tensioning of the mesh. **(B)** DZOH suture tied and elevating the vaginal cuff with the suture ends not yet trimmed and the vaginal cuff not completely closed. **(C)** Final appearance of the mesh with the sutures tied and the vagina closed off.

The vNOTES DZOH apical suspension technique requires that all 3 arms of the vaginal mesh be aligned on top of each other and attached together by threading the Prolene suture edges, extending from the apical arm serially through the anterior mesh piece and then the posterior mesh piece. All arms need to remain aligned and flat, with 1 sheet on top of the other, while ensuring that the Prolene suture is threaded at 1 cm from the free mesh end. The ends of the U-suture should be passed through these free ends of the anterior and posterior mesh pieces, respectively, and then tagged using a hemostat. The threads can either emerge (1) within the crux of the Y of the mesh, simplifying the suture tying, or (2) the threads can emerge behind the posterior arm (exiting from the mesh side connected to the posterior arm, away from the anterior arm). (Fig. 5) This latter positioning will enable retroperitonealization of the knot away from the vaginal-cuff tissue once it is tied. To enable tensioning, cuff elevation, and suture tying, the Prolene thread ends need to be held with a hemostat while emerging along 1 side of the vagina (preferably along the left vaginal fornix if the surgeon is right-handed).

The cuff closure is initiated next, using a 0-Vicryl suture started from the patient's right forniceal edge while suturing toward the midline. Another suture is then anchored to the left forniceal edge without closing that side of the cuff to enable introduction of the surgeon's finger or knot pusher into the space behind the cuff, enable guided tensioning of the vaginal apex.

The vaginal mesh can be tensioned in 2 different ways. An extracorporeal knot is made while all mesh layers remain attached. With 1 end of the suture held in place, the knot is pushed carefully past the vaginal-cuff closure site, thus mobilizing the crux of the mesh cephalad toward the sacrum. This will suspend the cuff, and the extent of elevation will guide the vaginal length. Then, the surgeon should assess the adequacy of the suspension and tie the knot securely. Compartment-specific adjustments may also be performed, if necessary, by rethreading and repositioning the Prolene suture ends in the anterior and posterior arms of the mesh, offering varied increased or reduced tension per compartment. Once securely tied, the excess suture edges should be trimmed carefully. If they are emerging behind the posterior arm, they should be tacked behind the apex of the cuff, pointing toward the pelvis and away from the vaginal-incision site, ideally placing the knot retroperitoneally and away from the vaginal tissue at the cuff.

A surgeon may also elect to tie the mesh directly and tension it digitally while avoiding a knot pusher. Another technique that limits undue tension on the mesh and suture during the procedure involves holding and elevating the cuff with a long Allis clamp. At the identified final apical location, the DZOH apical suspension suture is tied. Either method enables elevation of the vaginal cuff with a near anatomically correct axis, with the apical mesh tightening like an accordion due to the embedded Prolene suture. (Fig. 5)

Once adequately suspended, the remaining vaginal cuff is closed from the left to the right and both knots are tied in the midline, while avoiding incorporation of the mesh into the suture line. The suture line is preferably maintained 1–2 cm away from the Y-junction region of the mesh combination. Cystoscopy is performed at this point.

## Results

The SCP procedure described was performed effectively on cadaveric and live participants, wherein a list of steps was developed for so they could be reproduced. To assess the adequacy and longevity of the results, the steps were applied to a postmenopausal patient with symptomatic stage III POP with predominantly apical descent. She wanted to have surgical repair. She had agreed to undergo SCP with permanent mesh augmentation after extensive counseling. Her preoperative examination revealed a stage III uterine prolapse, with point C (POP-Q [Pelvic Organ Prolapse Quantification] scoring) at +5, and a total vaginal length (TVL) of 8 cm. Her postoperative course was uncomplicated. This patient was discharged in stable condition after a successful void trial on the same day of her surgery. She had adequate pelvic-floor support at 6 weeks, and 6 months, and at her 1-year postoperative follow-ups, with successful maintenance of apical suspension with point C at −8 and TVL at 8 cm.

## Discussion

There are potential advantages to performing a vNOTES SCP, compared to the conventional abdominal approach. vNOTES could optimize patient satisfaction via improvement of cosmesis, and decreased postoperative abdominal pain and risk of incisional hernia, with improved visualization and precision that is inherent to the use of laparoscopic instruments. Potential disadvantages include the limited data regarding vNOTEs' long-term outcomes and safety compared to the conventional approach, and a steeper learning curve secondary to the procedure's 2-D nature, novelty, and technical challenges. Lu et al. reported on the longest follow-up for vNOTES SCP, when they encountered both a 5.5% rate of prolapse recurrence and mesh exposure, similar to the rate reported at 2 years in abdominal SCP by Nygaard et al.^13,14^

The efficiency potentially gained in the technique described by the current authors can be attributed to both the use of surgical tackers and the lack of need for intracorporeal laparoscopic suturing.

Other techniques that require suturing involve an area exposed over the sacrum where stitches were placed as well as the mesh pathway that was completely dissected and exposed, requiring peritoneal-edge approximation prior to conclusion of the case. This can be avoided by varying the approach for access to the sacrum. Additionally, the tunneling technique, as described herein, or the use of the pure extraperitoneal approach—unlike the technique that involves peritoneal cutting and exposure—can preclude the need for the additional steps of reapproximating the peritoneal edges.^6^

Whereas many researchers have described direct suturing to the sacrum, use of a tacker improves ease and speed of attachment of the mesh while avoiding closure of the peritoneum above the mesh on the sacrum.^6,8^ Use of tackers has been described in various randomized controlled trials and was reported to result in similar long-term prolapse-support outcomes.^15–17^ Being 2-dimensional, laparoscopic intracorporeal suturing through the vNOTES port is reported to be more challenging than its robotic counterpart. Additionally, having the trocars placed in the vNOTES port a few centimeters apart limits the range of motion and impedes suturing due to the “chopstick” effect.^5^

Other researchers reported on a combination technique wherein both tackers and sutures were placed.^8^ Additional steps that improve efficiency include direct dissection of the anterior and posterior compartments followed by a nonlaparoscopic attachment of the mesh to the intra-abdominal aspect of both compartments. This precludes the need for laparoscopic dissection and suturing, enabling optimal intraoperative proficiency. This technique has been shown to produce a similar result to an entirely laparoscopic or robotically assisted approach.^3^

For developing the attachment sites of the mesh on the vaginal walls, Liu et al. described a technique similar to the current authors' methodology wherein hydrodissection helped identify the planes permitting for easy separation of the viscera off the fibromusclaris.^18^ A multicenter retrospective study published in 2022 focused on the route of hysterectomy and mesh attachment with no increased risk of complications with any specific route identified.^19^ A prospective study, also published by Liu et al. did not show any evidence of mesh-related complications in vaginal attachment of mesh in a follow-up period of up to 14 months.^4^ However, this could be secondary to the small sample size reported in their study.

In addition to efficiency, an increase in safety, improvement in precision, and circumvention of dissection-related injury have been reported in vNOTES SCP.^7^ This has been attributed to the improved visualization and accessibility of the presacral anatomy, compared to transabdominal laparoscopy. Furthermore, the technique described enables placement of the mesh in the proximity of the S-1–S-2 (below the intervertebral disc), which enables a more-anatomic alignment of the vagina, in turn, enabling equal distribution of pressures to the anterior and posterior vaginal walls.

Good et al. reported on the infectious safety and benefit of mesh attachment 1.5 cm below the L-5–S-1 intervertebral disc, a site less easily accessible when performing robotic SCP.^20^ This may help reduce compartment-specific recurrences due to pressure maldistribution, as has been noted with sacrospinous fixation where the anterior compartment is exposed to high pressures, while the posterior compartment may be more protected.^21^

Strengths of this approach include reproducibility on both cadaveric and live patients as well as the detailed anatomic depiction in illustrations (Figs. 1–5) and descriptions of the steps. Furthermore, the benefits of this work are inherent in the technique, including the surgeon-guided vaginal-mesh tensioning and cuff elevation, axis alignment, safety potentials, and efficiency in completing the steps of the case described. Limitations include the nature of vNOTES being both an emerging technique and technology, being based on only 2 surgeons' experience, and lack of more than 12-month long-term safety and efficacy follow-up of this SCP technique on the live patient.

## Conclusions

SCP may be performed successfully and efficiently by a vNOTES route, offering patients anatomically aligned apical suspension with compartment support that is adjustable intraoperatively based on the patient's needs. Well-designed prospective studies are needed to assess the suggested benefits long-term.
